# EHMTI-0182. Positive family history of migraine predisposes to a reduced migraineurs visual cortical reactivity

**DOI:** 10.1186/1129-2377-15-S1-E8

**Published:** 2014-09-18

**Authors:** G Coppola, M Bracaglia, D Di Lenola, G Di Ciaccia, C Di Lorenzo, V Parisi, F Pierelli

**Affiliations:** 1 Department of Neurophysiology of Vision and Neurophthalmology, G.B. Bietti Foundation-IRCCS, Roma, Italy; 2 Department of medico-surgical sciences and biotechnologies, Sapienza University of Rome Polo Pontino, Latina, Italy

## Background

In migraine, the genetic load can be seen as determining, on the one hand, a critical threshold for the disease development, and on the other hand, it may be responsible for interictal nervous system electrophysiological abnormality.

## Aim

Here, we were aimed to verify whether having a positive family history of migraine might influence cortical abnormal information processing in migraine patients.

## Method

We retrospectively collected 109 migraine patients from those reviewed who had visual evoked potentials (VEPs) recordings (6 blocks of 100 sweeps, 15 min of arc cheques, 3.1 repetition rate) and information about parental history of migraine. Neurophysiological recording data were compared with those of 42 healthy volunteers (HV) without a personal or family history of migraine.

## Results

We recruited 85 patients with and 24 without a positive family history of migraine. Patients who had one parent affected (mother or father) had significantly lower N75-P100 VEP amplitude blocks overall than those had no parents affected, the latter resulting not different from HV. Lack of VEP N75-P100 amplitude habituation was found in overall migraineurs compared with HV, irrespectively of whether they had a parent affected or not.

## Conclusion

These findings suggest that familial occurrence of migraine may predispose to a general reduced cortical reactivity to visual stimulation.

No conflict of interest.

